# Emotional labor and its influencing factors of clinical nurses: a cross-sectional study based on latent profile analysis

**DOI:** 10.3389/fpubh.2025.1496648

**Published:** 2025-02-28

**Authors:** Li Zhou, Wei Xiong, Mengmeng Hu, Hongjuan Chang

**Affiliations:** ^1^School of Medicine, Wuhan University of Science and Technology, Wuhan, Hubei, China; ^2^Department of Cardiology, Zhongnan Hospital, Wuhan University, Wuhan, Hubei, China

**Keywords:** clinical nurse, emotional labor, latent profile analysis, deep acting, surface acting, influencing factors

## Abstract

**Objective:**

The present study aimed to investigate latent classes of emotional labor among clinical nurses in China and analyze the influencing factors of emotional labor. With this as a reference, care managers can develop more targeted emotional labor intervention programs.

**Methods:**

This study enrolled 1,320 clinical nurses by using stratified random sampling from August to October 2023. A total of 1,279 nurses completed the following questionnaires: General Information Questionnaire, Emotional Labor Scale, Generalized Anxiety Disorder-7 scale (GAD-7), Patient Health Questionnaire-9 scale (PHQ-9), and Work–Family Conflict Scale. Based on the Emotional Labor Scale, the latent profile analysis (LPA) was used to explore the latent classes of nurses’ emotional labor. Then, univariate analysis and multivariate logistic regression analysis were used to explore the influencing factors of emotional labor.

**Results:**

Three latent classes were identified: generally low-level group (1.5%), high-level deep-acting group (17.2%), and high-level surface-acting group (81.3%). Male nurses were more likely to be in the generally low-level group than female nurses. Nurses with low scores on Work–Family Conflict Scale were more likely to be in the high-level deep-acting group than those with high scores. Compared with the nurses with very poor health, the nurses with very good health were more likely to be in the high-level deep-acting group, and nurses with general health were more likely to be in the high-level surface-acting group.

**Conclusion:**

The emotional labor of clinical nurses can be identified into three latent classes. Nursing managers can predict the latent classes of emotional labor based on characteristics such as demographic information and work–family conflict. Therefore, precise intervention can be implemented to reduce the consumption of internal resources and the occurrence of nursing adverse events caused by excessive emotional labor.

## 1 Introduction

Emotional labor refers to the employees’ disguise and management of behavior, facial expressions, tone of voice, and emotions by organizational rules (1). In 1983, the concept of emotional labor was formally introduced in Hochschild’s book “The Managed Heart: Commercialization of Human Feeling” (1). “Managing one’s emotions to create a publicly visible representation of the face and body.” To comply with job requirements, individuals may not be able to express their emotions according to their original intention, thereby generating emotional labor and exerting impacts on their physical and mental health (2). Emotional labor is not merely a component of nurses’ clinical practice but also an essential element of their professional development. Appropriate emotional labor can facilitate interpersonal interaction, enhance the nurse–patient relationship, increase patient satisfaction, and augment self-achievement. However, frequent emotional labor and poor emotional labor strategies can cause excessive depletion of internal resources, resulting in job burnout and thereby influencing the quality of nursing (3). Similar conclusions were obtained by a study involving 200 nurses focused on the correlation between emotional labor and mental health, as well as the mediating effect of job burnout in this relationship (4).

The strategies of nurses’ emotion management included active coping and emotional expression camouflage, which correspond to deep acting and surface acting in this study. Surface acting involves masking actual emotions such as using a fake smile to hide one’s true feelings, whereas deep acting involves trying to feel and express desired emotions such as modifying one’s feelings to suit the situation (5, 6). Surface acting can cause an inconsistency between facial expressions and internal feelings, reducing internal resources, making people more prone to emotional disorders, and reducing job satisfaction and performance (5–7). In other words, surface acting means that when nurses are in a foul mood, they will conceal their genuine feelings and feign the positive emotions demanded by the organization. Instead, deep-acting can make a person’s true feelings consistent with the organization’s desired emotional expression by changing an individual’s cognitive understanding of emotional events in depth (5). Grandey (7) pointed out that surface acting includes two important subscales: emotion hiding and emotion faking. Regarding emotion hiding, nurses often encounter numerous challenging situations in their daily work. For example, when dealing with critically ill patients, handling complicated nursing procedures, and coordinating tense doctor-nurse relationships, they frequently need to suppress the negative emotions that truly emerge in their hearts, such as anxiety, fatigue, and depression. For instance, after working for a long time under high intensity and then facing a series of urgent inquiries from newly admitted patients and their families, nurses must restrain their impatience caused by tiredness, maintain a calm and patient appearance on the surface, and prevent these negative emotions from being revealed to avoid interfering with the emotional state of patients and their families, thus ensuring the smooth progress of nursing services. This is a typical manifestation of emotion hiding, which requires nurses to possess strong emotional self-control ability and hide their true emotions beneath their professional appearance. As for emotion faking, it focuses on nurses’ active creation of positive emotional expressions that are contrary to their actual inner feelings. For example, during the night shift when there is a shortage of staff and a heavy workload, nurses still have to muster up their spirits, show friendly and amiable smiles, and give patients a sense of reassurance. Such behavior of emotion faking is an effort made by nurses to meet the professional requirements and satisfy the psychological needs of patients. However, excessive reliance on this strategy may bring psychological burdens to nurses themselves. In the subsequent research discussions, we will base on this to refine the analysis of nurses’ emotional labor, aiming to reveal how to optimize nurses’ emotional management strategies, improve the overall quality of nursing services, and enhance nurses’ professional well-being.

Meanwhile, the emotional labor management strategies of nurses are affected by multiple factors. Han et al. (8) found in their research that high emotional intelligence could reduce the surface acting of nurses and increase their use of deep-acting and natural expression strategies. Nurses with high emotional intelligence were more likely to adopt positive coping methods to adapt to the work environment and actively manage emotions through emotional regulation methods such as thinking and association. Andel et al. (9) in a longitudinal study found that the participation degree of nurses’ emotional labor was affected by their nursing environment. Patients’ uncivilized behaviors led to more surface-acting behaviors of nurses, and the influence of colleagues’ uncivilized behaviors on their surface-acting was moderated by hostile prejudice. Colleagues’ uncivilized behaviors only increased the surface acting of nurses with high hostile prejudice. However, the present research on the emotional labor of clinical nurses only remains on the surface, mainly focusing on the impacts of emotional labor on job burnout and mental health, while there are fewer applied studies on the latent category division of emotional labor as well as relevant intervention measures and management. Therefore, the intervention measures and coping suggestions put forward by the existing studies are rather empty. Emotional labor research needs to seek more effective and scientific theories and methods for allocating or managing emotional resources, so as to improve the physical and mental health levels of the vast group of emotional laborers (10).

Latent profile analysis (LPA), a new individual-centered statistical method, explains the relationships among external continuous variables through latent class variables and realizes the local independence among manifest variables (11). LPA judges the classification of individuals’ latent characteristics based on their response patterns to the manifest items, and uses objective statistical indicators to measure the accuracy and validity of the classification, so as to ensure the maximization of heterogeneity between groups and homogeneity within groups (12). With its high classification accuracy, latent profile analysis has been widely applied in fields such as management and psychology. Previous studies have reported analyses on the emotional labor of teachers and community workers. However, there are few reports on the latent profile analysis of the emotional labor of clinical nurses and its heterogeneity at home and abroad. A study (13) conducted in a general hospital in South Korea surveyed 207 nurses and found that among the different profiles of emotional labor strategies, nurses in the surface actor and high regulator profiles were more likely to experience a higher level of emotional exhaustion and had a stronger turnover intention compared to other types. It is worth noting that the “deep actor” profile was not found in this study. In view of this, this study aims to use latent profile analysis to classify the characteristics of emotional labor of clinical nurses in three Class-A tertiary hospitals in Hubei Province in China, explore the group differences and influencing factors among different categories, and provide a reference for targeted and precise intervention and prevention of excessive emotional labor among nurse groups according to the characteristics of different categories.

Research hypotheses:

*Hypothesis 1*: There is heterogeneity in the characteristics of the emotional labor of clinical nurses.

*Hypothesis 2*: There are differences in the influencing factors of emotional labor among different latent profile subgroups of clinical nurses.

## 2 Objects and methods

### 2.1 Study population

In this study, Hubei Province was divided into the north, central, and south regions according to the factors of urban economic level and regional spatial distribution. Then, a stratified random sampling method was used to randomly select one tertiary hospital in each region. The subjects were all clinical nurses who met the inclusion criteria in the three tertiary hospitals selected. Inclusion criteria: (1) on-the-job registered nurse; (2) working experience in the current department ≥1 year; (3) participants gave informed consent and participated voluntarily. Exclusion criteria: (1) standardized training, advanced study, and practice nurses; (2) nurses on leave during the survey; (3) those who suffered from family accidents or traffic accidents in the past 6 months. The study was approved by the ethics committee of Wuhan University of Science and Technology (approval No. 2023091). All participants gave informed consent and participated voluntarily.

### 2.2 Survey instruments

#### 2.2.1 General information questionnaire

The general information questionnaire was designed by the research team, including 11 items: age, gender, education level, marriage, job title, family relationship harmony degree, department, work intensity, frequency of night shift per month, sleep quality, and health status. The family relationship harmony degree was defined according to the frequency of bad emotions caused by family conflicts: disharmonious most of the time: the frequency is >10 times per year; generally harmonious, the frequency is 5–9 times per year; harmonious in most of the time, the frequency is 3–4 times per year; very harmonious, the frequency is 0–2 times per year.

#### 2.2.2 Emotional labor scale (ELS)

In the study, the emotional labor scale compiled by Grandey (14) and translated and revised by Luo et al. (15) was used. It includes the following three dimensions: surface acting (7 items), deep acting (3 items), and emotional expression requirements (4 items). Each item was scored using a 6-point Likert method, ranging from “strongly disagree” to “strongly agree,” and scored from 0 to 6. Factor analysis indicated that the three dimensions cumulatively explain 62.78% of the variation. The Cronbach’s *α* coefficient of the scale and the three dimensions were 0.838, 0.863, 0.858, and 0.751, respectively, indicating good reliability and validity.

#### 2.2.3 Generalized anxiety Disorder-7 scale (GAD-7)

The Generalized Anxiety Disorder-7 (GAD-7) (16) is a 7-item questionnaire to investigate the actual psychological status of the subjects in the past 2 weeks. Each item is scored on a scale of 0 (never) to 3 (almost every day), with a total score of 0 to 21. In this system, the higher the score suggests that the subjects have more anxiety: no anxiety, subjects scored 0–4 points; mild anxiety, subjects scored 5–9 points; moderate anxiety, subjects scored 10–14 points; severe anxiety, subjects scored 15–21 points. Cronbach’s alpha for the scale was 0.930.

#### 2.2.4 Patient health questionnaire-9 scale (PHQ-9)

Patient health questionnaire (PHQ-9) (17) was used to assess depressive mood in the past 2 weeks using four-point Likert-type scale. The higher the score suggests that the subjects are more depressed: mild depression, subjects scored 6–9 points; moderate depression, subjects scored 10–14 points; severe depression, subjects scored 15–19 points; extremely severe depression, subjects scored 20–27 points. Cronbach’s alpha for the scale was 0.913.

### 2.3 Data collection and quality control

Questionnaire Star, a web-based survey platform was used to make an electronic questionnaire. The team leader contacted the director of the nursing department of each hospital to explain the purpose of the study and the method of filling out the questionnaire. Then the two-dimensional code of the questionnaire was distributed to the nurses through the WeChat platform by the head of the nursing department. Each question was set as a required answer and can be submitted only when completed. Each IP address can only be filled in once. Two researchers checked the quality of the questionnaires and deleted the questionnaires with a response time of less than 10 min. Following the data collection, the research team meticulously examined the content and integrity of the questionnaires one by one and excluded the invalid questionnaires with incorrect filling, incomplete filling, and obvious rules in answers. A total of 1,320 questionnaires were collected in this study, and 1,279 were effectively recovered, with an effective recovery rate of 96.89%.

### 2.4 Statistical analysis

LPA was performed using Mplus software, version 8.3. Model adaptation was tested to select the best-fitting model (11). There were three criteria for evaluating the fitting indexes of the latent profile model: (1) the model with the smallest Akaike information criterion (AIC), bayesian information criterion (BIC), and sample size-adjusted Bayesian information criterion (aBIC) was the best; (2) the closer Entropy is to 1, the more accurate the classification is; (3) Two metrics, Lo–Mendell–Rubin (LMR) and Bootstrap likelihood ratio test (BLRT), were used to compare the fitting differences of latent class models. If the *p*-values of LMR and BLRT both reached the significant level, it indicated that the model with k categories fitted better than the model with K-1 categories. Analyses were conducted by SPSS 28.0 software (IBM Corp, Armonk, New York, USA). Categorical, normal distribution variables were presented as counts and percentages, mean and standard deviation, respectively. The Chi-square test was used to test categorical data. The one-way analysis of variance was used for the analysis of quantified independent normal distribution data between the three groups. Multivariate logistic regression analyses were performed for variables significantly associated with the results of LPA to explore influencing factors of emotional labor. A two-tailed *p* value <0.05 was considered statistical significance.

## 3 Results

### 3.1 Latent profile analysis of emotional labor in nurses

#### 3.1.1 Latent classes of nurses’ emotional labor

Table 1 shows adaptation indexes of different latent class models. As the number of latent classes increases, the AIC and BIC values gradually decrease, but the lowest value is not observed. The scree plot test (18) shows that when there are three latent classes, there is an obvious turning point, and the entropy value was 0.817, LMR, and BLRT were statistically significant (*p* < 0.001). By comprehensively comparing the adaptation indexes of the five models, we selected model 3 as the best-fitting model to analyze the emotional labor characteristics of clinical nurses.

**Table 1:** Adaptation indexes of different latent class models.

Model	AIC	BIC	aBIC	Entropy	LMR(P)	BLPT(P)	Category probility
1	21259.242	21290.165	21271.106	–	–	–	–
2	21015.242	21066.781	21035.016	0.713	<0.001	<0.001	0.184/0.816
3	20809.983	20883.137	20837.666	0.817	<0.001	<0.001	0.015/0.172/0.813
4	20706.379	20799.148	20741.971	0.795	<0.001	<0.001	0.014/0.751/0.130/0.105
5	20660.025	20773.409	20703.527	0.781	0.002	0.002	0.013/0.143/0.061/0.701/0.082

#### 3.1.2 Naming of latent classes

**Figure 1:**
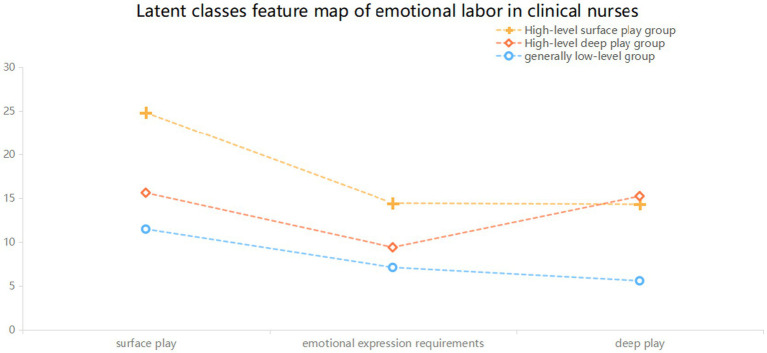
Latent classes feature map of emotional labor in clinical nurses.

Figure 1 shows the condition means of the three latent classes on three dimensions of the emotional labor scale. The classes were named based on the explicit characteristics of each dimension of the scale. The scores of nurses in the first class were at a low level in all dimensions, so this group was called the “generally low-level group.” The scores of nurses in the second class were at a high level in the deep-acting dimension, so this group was called the “high-level deep-acting group.” The scores of nurses in the third class were at a high level in the surface-acting dimension, so this group was called the “high-level surface-acting group.”

### 3.2 Univariate analysis among the three latent classes

Table 2 shows the comparison results among the three latent classes. The differences were statistically significant in gender, family relationship harmony, literacy level, work intensity, sleep quality, health status, generalized anxiety scale scores, patient health questionnaire-9 scale scores, and work–family conflict questionnaire scores. Specifically, significant differences are observed in gender distribution among different latent classes (*p* = 0.042). To be specific, males make up 2.3% of the total population and possess a relatively higher proportion of 11.1% in the group with generally low levels of emotional labor. In contrast, the proportions in the high-level deep acting group and high-level surface acting group are 2.7 and 2.1%, respectively. This implies that male nurses have a greater tendency to be in the group with generally low levels of emotional labor. Educational attainment also shows significant differences among different latent classes (*p* = 0.007). Undergraduates account for a relatively high proportion in the high-level deep acting group and high-level surface acting group (75.1 and 75.7%). It can be inferred that nurses with higher educational attainment are more prone to performing emotional labor at a higher level. The degree of family relationship harmony exhibits significant differences among different latent classes (*p* < 0.001). The proportion of very harmonious family relationships in the total population is 57.7%, and it reaches the highest proportion of 75.1% in the high-level deep acting group of emotional labor. This might suggest that a harmonious family relationship is advantageous for nurses to conduct deep-level emotional labor. Work intensity presents significant differences among different latent classes (*p* < 0.001). The proportion of very high work intensity in the total population is 17.98%, and it has relatively high proportions in the group with generally low levels of emotional labor and the high-level surface acting group, which are 27.8 and 19.0% respectively, while the proportion in the high-level deep acting group is only 11.4%. This indicates that high-intensity work may be unfavorable for nurses to conduct deep-level emotional labor. Sleep quality demonstrates significant differences among different latent classes (*p* < 0.001). The proportion of very good sleep quality in the total population is 4.1%, and it has a relatively high proportion of 10.3% in the high-level deep acting group of emotional labor. This reveals that good sleep quality may assist nurses in conducting deep-level emotional labor. Health status also shows significant differences among different latent classes (*p* < 0.001). The proportion of very good health status in the total population is 5.5%, and it has a proportion of 13.5% in the high-level deep acting group of emotional labor. This indicates that nurses with good health status are more likely to perform emotional labor at a higher level.

**Table 2:** Univariate analysis among the three latent classes.

Project	Total (*n* = 1,279)	Emotional labor lower level group (*n* = 18)	Emotional labor high-level deep play group (*n* = 185)	Emotional labor high-level surface play group (*n* = 1,076)	Inspection system calculates	*P value*
Age (year, x ± s)	33.66 ± 6.386	32.5 ± 6.537	33.82 ± 7.35	33.65 ± 6.207	69.046^2^	0.442
Gender					6.344^1^	0.042
Male	30(2.3)	2(11.1)	5(2.7)	23(2.1)		
Female	1,249(97.7%)	16(88.9)	180(97.3)	1,053(97.9)		
Degree of education					14.233^1^	0.007
Junior college and below	303(24.5)	11(61.1)	46(24.9)	256(23.8)		
undergraduate	961(75.1)	7(38.9)	139(75.1)	815(75.7)		
Postgraduate degree or above	5(0.4)	0(0.0)	0(0.0)	5(0.5)		
Marital status					3.621^1^	0.460
Single	195(15.2)	3(16.7)	29(15.7)	163(15.1)		
Married	1,056(82.6)	14(77.8)	155(83.8)	887(82.4)		
Widowed or divorced	28(2.2)	1(5.6)	1(0.5)	26(2.4)		
Family relationship					32.238^1^	<0.001
Very harmonious	738(57.7)	11(61.1)	139(75.1)	588(54.6)		
Harmonious in most of the time	418(32.7)	5(27.8)	36(19.5)	377(35.0)		
Generally harmonious	111(8.7)	1(5.6)	10(5.4)	100(9.3)		
Disharmonious in most of the time	12(0.9)	1(5.6)	0(0.00)	11(1.1)		
Department					11.243^1^	0.508
Internal medicine	449(35.1)	10(55.6)	67(36.2)	372(34.6)		
Surgical department	365(28.5)	2(11.1)	62(33.5)	301(28.0)		
Gynecology and obstetrics	101(7.9)	2(11.1)	16(8.6)	83(7.7)		
Pediatrics	72(5.6)	1(5.6)	6(3.2)	65(6.0)		
ICU	175(13.7)	2(11.1)	20(10.8)	153(14.2)		
Operating room	114(8.9)	1(5.6)	14(7.6)	99(9.2)		
Working intensity					30.977^1^	<0.001
Very large	230(17.98)	5(27.8)	21(11.4)	204(19.0)		
Bigger intensity	611(47.77)	10(55.6)	74(40.0)	527(49.0)		
Generally	431(33.70)	3(16.7)	86(46.5)	342(31.8)		
Smaller intensity	7(0.5)	0(0.00)	4(2.2)	3(0.3)		
Sleep quality					42.307^1^	<0.001
Very good	53(4.1)	0(0.0)	19(10.3)	34(3.2)		
Good	144(11.3)	2(11.1)	28(15.1)	114(10.6)		
Generally	635(49.7)	12(66.7)	101(54.6)	522(48.5)		
Bad	333(26.0)	4(22.2)	31(16.8)	298(27.7)		
Very bad	114(8.9)	0(0.0)	6(3.2)	108(10.0)		
Health condition					57.563^1^	<0.001
Very good	70(5.5)	0(0.00)	25(13.5)	45(4.2)		
Good	318(24.9)	8(44.4)	67(36.2)	243(22.6)		
Generally	716(56.0)	8(44.4)	83(44.9)	625(58.1)		
Bad	139(10.9)	2(11.1)	8(4.3)	129(12.0)		
Very bad	36(2.8)	0(0.00)	2(1.1)	34(3.2)		
Anxiety scale score	6.78±5.112	4.56±4.190	4.19±4.184	7.26±5.131	99.420^2^	<0.001
Depression module scale score	7.95±5.878	4.33±5.258	4.52±4.537	8.60±5.869	142.607^2^	<0.001
Work–family conflict scale score	55.64 ± 9.433	56.06 ± 10.984	48.75 ± 9.520	56.82 ± 8.876	286.439^2^	<0.001

1 Chi-square value χ^2^.

2 F value.

Regarding the scores of the Generalized Anxiety Disorder scale and the depression module scale in the health questionnaire, significant differences are found in the scores of these two scales among the three latent classes (*p* < 0.001). Nurses with lower scores of anxiety and depression are more likely to be in the high-level deep acting group of emotional labor, suggesting that emotional state has an impact on the level of nurses’ emotional labor. The scores of the work–family conflict questionnaire display significant differences among different latent classes (*p* < 0.001). The scores in the group with generally low levels of emotional labor and the high-level surface acting group are relatively high (56.06 ± 10.984 and 56.82 ± 8.876), indicating that work–family conflict may affect the depth of nurses’ emotional labor.

However, no significant differences are detected in age, marital status, and department among the three latent classes (*p* = 0.442, *p* = 0.460, *p* = 0.508), signifying that age, marital status, and department factors have little influence on the classification of nurses’ emotional labor latent classes. These results are presented in Table 2.

**Table 3:** The assignments of variables.

Variable	assignment method
The quality of sleep	Very good = 1; good = 2; average = 3; poor = 4; very poor = 5
Gender	Male = 1; female = 2
Family relationship	Very harmonious =1; most of the time harmonious =2; generally =3; most of the time discordant = 4; very discordant = 5
Education level	Secondary school =1; College = 2; Undergraduate = 3; Master’s degree and above =4
Work intensity	Very large = 1; larger = 2; general = 3; smaller = 4; very small = 5
Health status	Very good health = 1; good = 2; average = 3; poor = 4; very poor = 5
Work–family conflict scale score	The measured value

### 3.3 Multivariate regression analysis of different classes

Multiple-factor non-conditional logistic regression analysis was conducted with the latent classes of nurses’ emotional labor as dependent variables, statistically significant factors in the univariate analysis as independent variables, and continuous variables as covariates. The method of assigning values to independent variables can be found in Table 3. In the multivariate regression analysis of different class in comparison with the high-level deep acting group and high-level surface acting group, male nurses are more inclined to be in the group with generally low levels of emotional labor (OR = 0.045, 95%CI: 0.011–0.184; OR = 0.042, 95%CI: 0.015–0.117); when contrasted with the group with generally low levels of emotional labor, nurses with very good health status are more likely to be categorized into the deep acting group, and nurses with average health status are more likely to be grouped into the surface acting group (OR = 37.803, 95%CI: 1.054–1355.613; OR = 8.551, 95%CI: 1.802–40.584); compared with the group with generally low levels of emotional labor, nurses with high scores of work–family conflict are less likely to be classified into the high-level deep acting group (OR = 0.911, 95%CI: 0.873–0.951). The results are presented in Table 4.

**Table 4:** Multivariate regression analysis of different class.

Item	*β* value	Standard error	Wald x^2^	*P* value	OR value	95% CI
High-level deep play group vs. generally low-level group						
Intercept	6.891	8.863	0.604	0.437		
Work–family conflict score	−0.093	0.022	17.774	0.000	0.911	0.873–0.951
Male nurse	−3.094	0.715	18.705	0.000	0.045	0.011–0.184
Health status						
Very good	3.632	1.826	3.956	0.047	37.803	1.054–1355.613
Good	1.739	1.042	2.886	0.089	5.691	0.766–42.314
General	2.185	0.992	4.850	0.028	8.889	1.272–62.126
Poor	1.437	1.151	1.560	0.212	4.210	0.441–40.179
High-level surface play group vs. generally low-level group						
Intercept	7.400	8.452	0.766	0.381		
Male nurse	−3.165	0.518	37.347	0.000	0.042	0.015–0.117
Health status						
Very good	2.931	1.722	2.897	0.089	18.746	0.642–547.768
Good	1.423	0.827	2.958	0.085	4.150	0.820–21.002
General	2.146	0.795	7.295	0.007	8.551	1.802–40.584
Poor	1.462	0.949	2.376	0.123	4.315	0.672–27.695

The high-level deep play group and the high-level surface play group are used as reference groups for the generally low-level group. Males are used as a reference for females; very poor health is used as a reference.

## 4 Discussion

### 4.1 The emotional labor of clinical nurses with different demographic characteristics is heterogeneous

The study showed that the emotional labor score of 1,279 nurses in three tertiary hospitals was 50.74 ± 9.305, which was in the upper middle level and consistent with the study by Hu (19). Nurses accounted for 1.41, 14.46, and 84.13% of the generally low-level group, high-level and deep-acting group, and high-level and surface-acting group, respectively. Most nurses’ emotional labor was in the high-level and surface-acting group, which was different from the results of studies by Lv et al. (20) and Bian et al. (21). The reason for the difference may be that the research subjects and latent classes were different. Most of the subjects in previous studies were front-line community workers and teachers, other groups. Everyone Fouquereau et al. (22) put forward in the study that researchers relied on LPA to identify subpopulations of workers characterized by distinct configurations of hiding feelings, faking emotions, and deep acting. However, the present study was on nurses in clinical departments. When nurses are faced with high pressure and overload work pressure, surface acting is the first choice. Nurses in the high-level surface-acting group were primarily those with high work intensity, general sleep quality, general health status, and mild anxiety and depression. They were confronted with excessive work pressure, overtime work, shift work, and a growing phenomenon of workplace bullying, which resulted in low morale, emotional exhaustion, and reduced happiness (23). All of these circumstances might compel nurses to feign happiness to meet organizational expectations, thereby generating surface acting (24). Nurses in the high-level deep-acting group were primarily those with general work intensity, harmonious family relationships, good sleep quality, low anxiety scores and free from anxiety. They were in a relaxed and pleasant working atmosphere and would satisfy the expectations of the organization from the heart. Thus, their emotional expression was natural and true (14).

### 4.2 The influencing factors of latent classes of emotional labor in nurses

#### 4.2.1 Male nurses were more likely to be in the generally low-level group than female nurses

Logistic regression analysis showed that male nurses were more likely to be in the generally low-level group than female nurses (high-level and deep-acting group vs. generally low-level group: OR 0.045, 95% CI 0.011–0.184; high-level and surface-acting group vs. generally low-level group: OR 0.042, 95% CI 0.015–0.117). This was consistent with the studies of Yao et al. (25) and Gulsen et al. (26). Due to the influence of physiological mechanisms and social factors, male nurses generally have stronger stress resistance than females and tend to exhibit more rationality and low-level emotional labor in the workplace (26). Male nurses are often assigned to high-risk and high-pressure departments, such as emergency departments, operating rooms, ICU, and psychiatric departments, where they need to possess stronger anti-stress abilities and the capacity to rationally handle problems (27). Meanwhile, in China, men usually play the role of the breadwinner in the family (28), and this social expectation further requires them to remain calm and composed when solving problems. Female nurses exhibit superior skills in managing and suppressing genuine emotions, regulating their own emotions in response to external emotional cues, and possessing better emotional regulation capabilities. Emotional labor is particularly central to some occupations. For example, Gray (29) demonstrated that emotional labor is a core component of nurses’ role in making patients feel safe and comfortable. In other words, emotional labor is an almost invisible bond that the nurse cultivates with the patient (1). Therefore, it is necessary for male nurses to develop diverse emotional labor strategies to ensure the smooth progress of nurse–patient communication. Thus, it is proposed that a working group comprising male nurses should be established within the hospital, and emotional expression workshops should be held regularly to encourage male nurses to reveal their genuine emotions and guide them to undertake more positive emotional labor. In 2014, the Chinese Nursing Association (30) established a male nurse working group, which encourages male nurses to participate in specialized nurse training courses, scientific research classes, and English salons, providing sufficient platforms and motivating male nurses to develop in a specialized direction. The male nurse team, by virtue of their natural physical strength, endurance advantages, and excellent emergency response capabilities, can actively engage in various volunteer service activities such as first aid science popularization youth public welfare activities, marathon volunteering, and unpaid blood donation to contribute to society. More career planning training and career development opportunities can enhance the professional recognition of male nurses, strengthen their social influence, consolidate their professional image, and enable male nurses to achieve their professional accomplishments more confidently and stably. While serving others, the male nurse working group also regularly organizes various public welfare publicity activities to exchange clinical experiences, career confusions, and work insights. Kharatzadeh et al. (31) conducted emotion regulation training for nurses in the intensive care unit, including psychoeducation, progressive muscle relaxation, acceptance and tolerance of emotional responses, cognitive reappraisal, problem-solving, and interpersonal skills, which effectively alleviated the nurses’ depression, anxiety, burnout, and improved their cognitive coping strategies. Meanwhile, moderate exercises and relaxation activities, such as walking and weight training, can assist in releasing stress and enhancing emotional management.

#### 4.2.2 Compared with the nurses with very poor health, the nurses with very good health were more likely to be in the high-level and deep-acting group

Logistic regression analysis showed that compared with the nurses with very poor health, the nurses with very good health were more likely to be in the high-level and deep-acting group (high-level deep-acting group vs. generally low-level group: OR 37.803, 95% CI 1.054–1355.613), and nurses with general health were more likely to be in high-level surface-acting group (high-level surface-acting group vs. generally low-level group: OR 8.551, 95% CI 1.802–40.584). This was consistent with the studies of Xie et al. (32). Health is not only the absence of disease or infirmity, but also an intact state of physical, mental, and social adaptation. Mental health refers to the ability of individuals to maintain a good mental state and adaptability when confronted with various challenges and pressures. Good mental health has a positive predictive effect on deep acting. Deep acting represents a form of self-regulation emanating from within the heart, aiming to achieve the consistent emotional expression demanded by the circumstances. It is a consistent emotional expression strategy shaped through self-regulation (33). This strategy is conducive to both physical and mental health as well as organizational effectiveness. It also encourages individuals to be thoughtful of others in a negative emotional setting, decrease dissatisfaction with adverse events and characters, and thereby reduce their internal discomfort. However, when nurses with poor health conditions experience pain, fatigue, psychological stress, and other factors that lead to low work efficiency and an inability to fully engage in work, and when this affects work efficiency and quality, it can be referred to as invisible absent nurses or impaired health productivity (34). This not only have difficulty in guaranteeing their personal physical and mental health, but also may increase the risk of patient safety and lower the quality of nursing. Such nurses frequently adopt surface acting and negative emotion management strategies to handle work and study, lacking sincerity, thereby creating a false organizational atmosphere and greatly reducing work efficiency. Everyone Fouquereau (22) point out that the wider the gap between organizational display rules and workers’ genuine emotions, the less inclined workers may be to identify with their job, leading them to experience reduced levels of job satisfaction and an increased sense of psychological disconnection from their work (35). Such feelings of inauthenticity may make them less likely to experience healthy levels of psychological detachment and more likely to have sleeping problems given the links between surface acting and rumination (36). Therefore, nursing managers should pay attention to the physical and mental health of nurses, enable them to enhance their awareness of self-care, and allow them to ask for leave promptly and collaborate with the treatment when they fall ill. Meanwhile, hospitals can enhance the efficiency of nursing resources by establishing a flexible nurses’ pool, appropriately increasing reserve nurses of various levels and different specialties, and adopting diversified scheduling approaches.

#### 4.2.3 Nurses with low scores on the work–family conflict scale were more likely to be in high-level and deep-acting groups than those with high scores

Logistic regression analysis showed that nurses with high scores on work–family conflict scale were less likely to be in the high-level deep-acting group than those with low scores (high-level deep-acting group vs. generally low-level group: OR 0.911, 95% CI 0.873–0.951). This was consistent with the studies of Liao (37). Work and family affect and restrain each other. When a conflict arises between them, nurses often require more energy to achieve balance and are more prone to experiencing work fatigue (4), which reduces the possibility of deep acting. For instance, backbone nurses endure greater pressure from teaching, management, scientific research, and family, leading to an intensified conflict between work and family, excessive depletion of their resources, and a propensity for them to adopt surface acting (25). Additionally, nurses frequently obtain additional work tasks through phone or WeChat during non-working hours (38). This extra work not only fails to increase job productivity (39), but also reduces job satisfaction and happiness, thereby causing job burnout. Hence, nursing managers should take effective measures to enrich incentive policies to the individual circumstances of nurses (25). Firstly, if nurses are obliged to work overtime, leaders are supposed to offer appropriate compensation, such as bonuses and family-related vacations. Secondly, the two-way communication (39) between nurses and managers enables managers to have a better understanding of each nurse’s specific working and family circumstances, and offer support and assistance to them. Moreover, regular stress reduction training sessions and emotional management workshops are arranged to direct nurses to accurately adopt emotional expression strategies and enhance the chances for profound emotional expression, thereby facilitating nurses to offer superior services to patients and their families.

### 4.3 Limitations and perspectives for future research

The current results possess certain limitations that warrant attention in subsequent research. Firstly, with respect to the research design, the present study is founded on cross-sectional data, which precludes the demonstration of dynamic alterations and critical turning points in nurses’ emotional labor. Future investigations should adopt a longitudinal study design to more comprehensively explore the developmental trajectory of nurses’ emotional labor and population heterogeneity. Secondly, nurses’ emotional labor exhibits variability across different scenarios. The emotional labor of nurses in diverse nursing contexts is characterized by the nature of interactions and emotional requirements. Deep acting is of crucial significance in delivering empathetic patient care, especially in palliative settings, as it facilitates nurses to establish genuine emotional connections with patients. In contrast, surface acting proves essential when interacting with families during challenging periods, enabling nurses to maintain a composed and appropriate outward appearance. Within nursing teams, effective emotional regulation serves as a cornerstone for maintaining team cohesion and fostering collaboration. During crises, the capacity to suppress emotions is necessary for formulating rational and clear responses, thereby highlighting the importance of nurses’ resilience. Overall, adeptly managing these emotional demands is fundamental to nursing proficiency and exerts a profound impact on both nurses’ well-being and the quality of patient care. It represents a complex and multifaceted dimension of the nursing profession that necessitates continuous scrutiny and enhancement. The current article lacks precision in the exploration from a situational perspective. Future research could be directed toward investigating the characteristics of clinical nurses’ emotional labor. Thirdly, concerning sample selection, the study merely surveyed nurses from three tertiary general hospitals in Hubei Province, China. Our study was confined to nurse employees in Hubei Province, China, without incorporating nurses from other regions or specialized hospitals. This lack of sample diversity restricts the generalizability of the research findings. Consequently, it remains uncertain whether the latent profiles identified in this study are specific to Chinese culture, particularly given that previous studies have indicated that the outcomes of emotional labor performance may differ across cultures. Fourthly, in this study, a self-reported emotional labor scale was utilized, which might introduce research bias. Future research could integrate qualitative and quantitative research methodologies to explore the influencing factors of clinical nurses’ emotional labor from multiple perspectives and dimensions.

## 5 Conclusion

Based on potential profile analysis, this study identified three potential categories of nurses ‘emotional labor through latent profile analysis, which provided a new perspective for understanding the diversity of nurses’ emotional labor. It also verified the research hypothesis put forward at the beginning of the article—that there is heterogeneity in the characteristics of emotional labor among clinical nurses. Meanwhile, the emotional labor of different latent profile subgroups is influenced by factors such as gender, health status, and work–family conflict, which further confirms that there are differences in the influencing factors of emotional labor among different latent profile subgroups of clinical nurses. Therefore, in clinical practice, the results provide a basis for nursing managers to predict emotional labor characteristics according to nurses’ demographic data and work–family conflicts, so as to implement precise intervention measures. Targeted recommendations is needed that the study expects nursing managers to provide precise interventions on emotional labor characteristics of clinical nurses in order to improve nurses ‘professional identity and job happiness index. However, this research is subject to certain limitations due to the relatively restricted sample source range and the adoption of the cross-sectional study method. Subsequent research can utilize multi-center large-sample survey methods and combine them with longitudinal study designs to further explore the trajectory and demographic heterogeneity of nurses’ emotional labor.

## Data Availability

The raw data supporting the conclusions of this article will be made available by the authors, without undue reservation.
